# Digital twins for plant–microbe interactions: Gap finding and filling

**DOI:** 10.1016/j.xplc.2026.101871

**Published:** 2026-04-18

**Authors:** Seonghan Jang, Seon-Kyu Kim, Jin-Soo Son, Choong-Min Ryu

**Affiliations:** 1 Molecular Phytobacteriology Laboratory, Infectious Disease Research Center, Korea Research Institute of Bioscience and Biotechnology (KRIBB), Daejeon 34141, South Korea; 2 Genomic Medicine Research Center, Korea Research Institute of Bioscience and Biotechnology (KRIBB), Daejeon 34141, South Korea

## Main text

Digital twins dynamically link virtual models with real-time data to enable predictive optimization and “what-if” simulations. Their recent adoption in the life sciences has revealed both strong appeal and substantial challenges, as biological systems are dynamic, nonlinear, and only partially observable. Even so, digital twins are increasingly recognized as a forward-looking priority in precision medicine (Laubenbacher et al., 2021; Niarakis et al., 2024). In plant science, however, their application remains nascent. Yet plant systems provide a particularly favorable testbed: unlike human-centered models, key variables can be precisely manipulated under controlled conditions, such as in growth chambers and plant factories, and destructive sampling is feasible. We therefore envision plant–microbe digital twins as immediately useful in studies of immunity and symbiosis, breeding for disease resistance, cultivation management in controlled environments, and field-scale crop protection.

**Figure 1:**
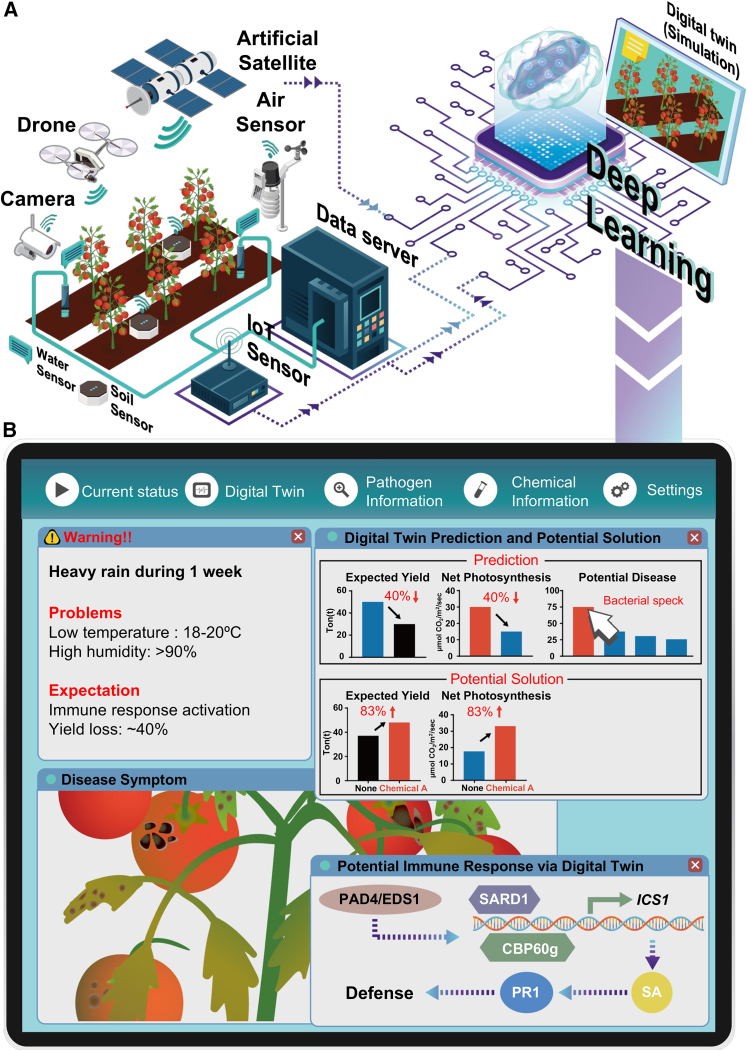
Conceptual roadmap and applications of plant–microbe digital twins. **(A)** Data acquisition and transmission for digital twin prediction. An artificial satellite monitors regional climate variation, while Internet-of-Things (IoT) sensors continuously collect environmental parameters such as air humidity, solar radiation, and atmospheric CO_2_ concentrations. Infrared cameras track leaf temperature and photosynthetic efficiency, and soil sensors measure pH, moisture content, electrical conductivity (EC), and NPK levels. Water-quality sensors assess irrigation parameters such as pH and ion concentrations. Drones capture high-resolution phenomic dynamics of tomato plants across developmental stages, and all data are transmitted in real time to a central server. AI and deep-learning algorithms integrate these datasets to drive digital twin simulations, enabling the prediction of physiological disorders, disease outbreaks, and yield reductions, and facilitating the derivation of optimal management strategies. **(B)** Example of a digital-twin-based predictive simulation. As an illustrative case, the digital twin simulates climatic conditions for the coming week based on acquired data, incorporating a scenario of heavy rainfall, low temperature, and high humidity. Under these simulated conditions, the system predicts potential disease outbreaks, reduced net photosynthetic rates, and decreased yield. The analysis recommends applying chemical A as a potential mitigation strategy and visualizes predicted pathogen dynamics and host immune-response pathways, thereby providing farmers with data-driven guidance for timely and informed decision-making. Pipeline modules are annotated with technological readiness level (TRL), biological readiness level (BRL), and biological credibility (BC) to indicate system maturity.

Although progress is constrained by environmental variability and fragmented data infrastructures, early studies already demonstrate feasibility. These span plant-scale frameworks integrating multi-omics data, orchard-scale citrus twins, and airflow simulations in plant factories (Jeong et al., 2022; Kim and Heo, 2024; Ganapathysubramanian et al., 2025). Together, these efforts suggest that digital twins could become indispensable tools for sustainable agriculture (Figure 1). Realizing this potential will require close integration of molecular plant biology and computational modeling, allowing digital twins to evolve from descriptive representations into predictive, actionable systems.

## Modeling plant–microbe interactions: From digital data to the real world

### Plant–pathogen dynamics and immune networks

In plant pathology, digital twins can serve as high-fidelity surrogates that link molecular responses to field-level outcomes (Ganapathysubramanian et al., 2025). Such models should integrate immune signaling, physiological processes, and infection dynamics while being calibrated with imaging platforms or biosensors capable of detecting early biotic stress. Internet-of-Things devices that monitor soil and plant canopies, combined with aerial observations, can support site-specific prediction (Kim and Heo, 2024), enabling both early outbreak forecasting and virtual testing of alternative interventions.

The central hurdle is translating molecular knowledge into a computable form. Systems biology frameworks, including Boolean networks and ordinary differential equations, can represent pattern-triggered immunity and effector-triggered immunity thresholds. When parameterized with time-series transcriptomic data, such models can simulate defense priming, hormone-regulated trade-offs, and the timing and amplitude of downstream responses. Operationally, an immune digital twin requires mapping hub genes from multi-omics datasets and converting them into mathematical modules. To function dynamically, these modules must be coupled with non-destructive, real-time sensor streams, such as hyperspectral imaging, so that parameters can be continuously recalibrated. This approach would allow researchers to test intervention timing *in silico* before field implementation.

### Symbiosis and phytobiomes: Metabolic exchange

Digital twins could also model beneficial plant–microbe relationships at the community level. This requires the explicit representation of nutrient and signaling exchange in virtual environments, coupled with iterative updates from plant observations. Advances in AI-driven modeling suggest that multi-omics integration and machine learning can support *in silico* microbial replicas (Helmy et al., 2024). An ideal plant–microbiome twin would therefore integrate metagenomic and metabolomic data to predict how changes in community composition influence plant traits and to forecast biocontrol outcomes.

Flux balance analysis provides a practical framework for simulating these metabolic interactions. A digital twin could incorporate genome-scale metabolic models in which root exudate profiles act as dynamic constraints that vary with photosynthetic rates. This would enable quantitative prediction of how specific microbial groups, such as nitrogen fixers, are recruited under stress (Yang et al., 2025). By centering metabolism, these frameworks convert broad microbiome effects into testable outputs, such as biomass accumulation, and position digital twins as platforms for designing synthetic consortia prior to field deployment (Helmy et al., 2024). When calibrated with time-series datasets, such models may also support probabilistic forecasts of community dynamics, allowing management strategies to be prioritized before experimental validation.

## Application of digital twins

### Disease control

Agricultural digital twins have immediate relevance for disease and pest management. By integrating environmental monitoring with plant health signals, these systems can forecast the timing and location of outbreaks and enable *in silico* comparisons of intervention strategies to maximize efficacy while reducing chemical inputs (Ganapathysubramanian et al., 2025). However, many current implementations function mainly as advanced monitoring dashboards. To qualify as true digital twins, systems should meet three minimum criteria: (i) continuous data assimilation that links the physical system to its virtual counterpart; (ii) predictive modeling that forecasts temporal biological outcomes rather than merely describing current states; and (iii) the capacity for *in silico* “what-if” simulations to compare intervention strategies. Without quantitative benchmarks—such as before-and-after comparisons of disease incidence or intervention efficiency—these systems remain proofs of concept. Establishing such metrics is therefore essential for digital twins to become evidence-based decision-support tools.

### Enhancing plant performance through microbiome engineering

Beyond plant protection, digital twins could improve crop productivity and resilience. In controlled environments, plant-factory twins based on computational fluid dynamics and calibrated with sensor data can optimize microclimate management by predicting airflow patterns (Jeong et al., 2022). At the orchard scale, individualized mandarin tree models have already guided micro-precision interventions, pointing toward “personalized agriculture” (Kim and Heo, 2024). Digital twins may also serve as platforms for microbiome engineering, enabling *in silico evaluation of* synthetic consortia or engineered strains and their effects on crop performance. This capability could streamline the design of beneficial plant–microbe partnerships (Helmy et al., 2024).

## Strategies for developing adaptive digital twins

### Key barriers to developing plant–microbe digital twins

Developing practical plant–microbe digital twins requires progress on three major challenges.

First, biological variability and genotype × environment × management (G×E×M) interactions introduce pervasive uncertainty. Unlike engineered machines, plants exhibit phenotypic plasticity and respond unpredictably to fluctuating conditions and biological noise. A robust twin therefore cannot rely solely on deterministic equations; it must represent biological components as dynamic agents with autonomous feedback loops. Perennial crops such as citrus illustrate this clearly: tree-to-tree structural heterogeneity necessitates repeated 3D canopy mapping (Kim and Heo, 2024). Annual row crops face a different limitation: a shortage of synchronized, multi-environment phenotyping datasets required to calibrate genotype-specific models (Cai et al., 2025).

Second, data integration across scales remains challenging. A true twin must couple processes spanning molecular pathways to ecosystem dynamics. The rhizosphere represents a critical blind spot, despite being the primary site of many key plant–microbe interactions. Moreover, models must be sensitive enough to capture low-abundance taxa that may exert disproportionate effects on immunity but are often overlooked. Real-time, multi-scale modeling also remains computationally demanding (Ganapathysubramanian et al., 2025). These limitations parallel the incomplete understanding of immune dynamics that constrains medical digital twins (Laubenbacher et al., 2021; Niarakis et al., 2024).

Third, interoperability and standardization are major bottlenecks. Data are frequently siloed across breeders, pathologists, agronomists, and farmers in incompatible formats (Guo et al., 2024). Broader adoption will therefore depend on unified interoperability frameworks and community-agreed standards. Although public multi-omics repositories are increasingly available, their integration into standardized digital twin architectures remains limited.

To address environmental variability and nonlinear dynamics, we advocate graph neural networks (GNNs) as a scalable framework for integrating heterogeneous, multi-scale plant–environment data. GNNs can link genomic regulatory networks, real-time soil sensor streams (e.g., moisture, temperature, and pH), and spatiotemporal weather inputs within a unified predictive graph (Przymus et al., 2025). This structure supports continuous updating, as incoming Internet-of-Things and meteorological data can refine node embeddings through localized message passing without costly full-model retraining. For plant–microbiome systems, recent work shows that GNN architectures can integrate genome-scale metabolic models with graph-based learning to predict microbial community dynamics and cross-feeding interactions (Hasibi et al., 2024). Coupled with real-time weather data, machine-learning models such as artificial neural networks and random forests can predict field disease severity with high accuracy (e.g., R^2^ > 0.93 for wheat pathogens; Bijlwan et al., 2025). Long short-term memory networks further capture temporal disease and stress dynamics, enabling proactive scheduling of interventions.

### Validation and standardization

A practical digital twin requires explicit validation criteria that biologists can apply across crops and applications. High-temporal-resolution multi-omics and phenomics datasets collected under defined perturbations should serve as ground truth for calibration. Evaluation metrics also require standardization—for example, root-mean-square error for yield prediction and the F1 score for early disease classification. Public repositories and coordinated field trials using synthetic consortia could provide shared benchmarks, thereby improving reproducibility, scalability, and cross-site comparability. To ensure usability, data sharing should follow FAIR principles, include consistent metadata, provide versioned releases, and specify clear data-use licenses or embargo policies so that independent groups can recalibrate and validate models without ambiguity.

In practice, validation should be task-specific. Disease forecasting should be evaluated using precision–recall metrics and rigorous cross-year validation. Yield prediction requires cross-site testing across diverse environments. Microbiome engineering should be evaluated through independent assessments of community stability across both greenhouse and field conditions.

### Hybrid modeling and orchestration

Several principles can guide the maturation of these systems. First, the timeline problem must be addressed. Without explicit temporal models of infection onset, immune responses, or symbiotic stabilization, digital twins remain descriptive rather than predictive (Niarakis et al., 2024; Ganapathysubramanian et al., 2025). Second, we advocate a hybrid approach—physics-informed machine learning—in which deep learning models are constrained by biological laws such as mass balance and growth kinetics. Generalization across sites and seasons must also be explicitly addressed. Models trained in one region should adapt to new climates or soils using limited local data, making transfer learning and meta-learning particularly attractive. Likewise, the inherent variability of agricultural systems calls for multi-environment training. Federated learning could enable research stations and farms to collaboratively train predictive models across diverse G×E×M contexts without centralizing sensitive or proprietary data. Third, a microbiome-centered perspective is essential for dynamically simulating metabolic exchange and linking molecular mechanisms with management decisions across scales (Escriba-Gelonch et al., 2024).

## Concluding remarks and perspective

Digital twins for plant–microbe interactions remain at an early stage; however, their potential impact on agriculture is substantial. By providing predictive, data-driven representations of plant health, they could bring unprecedented precision to crop management. Early demonstrations—from plant-factory airflow models to individualized orchard interventions—already support their feasibility (Jeong et al., 2022; Kim and Heo, 2024). The longer-term vision is a dynamic counterpart for each crop, or even each plant, capable of guiding tailored interventions in a manner analogous to personalized medicine (Laubenbacher et al., 2021). Achieving this goal will require robust infrastructure, rigorous validation, and sustained collaboration across plant biology, microbiology, engineering, and data science.

Although many current applications remain conceptual, the convergence of sensing technologies, AI, and biological modeling suggests that digital twins may ultimately become trusted advisers for crop protection, yield optimization, and microbiome engineering (Ganapathysubramanian et al., 2025). Progress will depend on explicitly linking molecular processes—from pattern-triggered immunity and effector-triggered immunity signaling to root exudate–microbe interactions—so that digital twins function not merely as engineering abstractions but as testable hypotheses in plant biology. Near-term priorities should therefore include standardizing multi-scale data collection, validating predictive models in controlled environments, and translating core molecular processes into computable modules. Longer-term goals should shift toward field-scale generalization across diverse G×E×M settings, microbiome engineering, and ultimately the development of individualized crop models that act as predictive engines for personalized agriculture.

## Funding

This work was supported by the Biomedical Technology Development Program of the National Research Foundation (NRF), funded by the Ministry of Science and ICT (grant nos. RS-2023-00219213 and RS-2024-00336247); the Rural Development Administration (project no. RS-2025-02216026); and the KRIBB Research Initiative Program, Republic of Korea.

## Acknowledgments

No conflict of interest declared.

## Author contributions

Conceptualization, S.J., S.-K.K., and C.-M.R.; visualization, J.-S.S.; supervision, C.-M.R.; writing – original draft, S.J. and S.-K.K.; writing – review and editing, C.-M.R.

## Declaration of generative AI and AI-assisted technologies in the writing process

During the preparation of this work, the authors used generative AI technologies to assist with language refinement and the generation of conceptual illustrations. The authors reviewed and edited all content as necessary and take full responsibility for the content of this publication.
